# Delayed depolarization and firing behavior of human motoneurons during voluntary muscle contractions

**DOI:** 10.3389/fnhum.2013.00793

**Published:** 2013-11-18

**Authors:** Lydia P. Kudina, Regina E. Andreeva

**Affiliations:** Department of Bioinformatics, Institute for Information Transmission Problems (Kharkevich Institute), Russian Academy of SciencesMoscow, Russia

**Keywords:** human motor control, voluntary muscle contraction, motoneuron firing behavior, double discharges, delayed depolarization

## Introduction

The firing behavior of motoneurons is governed by the interaction between the intrinsic properties of motoneurons and the synaptic inputs that they receive. In particular, long-lasting after-hyperpolarization (AHP) following each motoneuron spike and thus decreasing motoneuron excitability immediately after spike was found to be a key mechanism in controlling of interspike interval (ISI) duration (Eccles et al., 1958; Kernell, 1965). As a result, motoneurons commonly fire single discharges at rather low firing rates, especially during gentle voluntary muscle contractions and postural tasks. However, some motoneurons, under conditions of weak synaptic drive, can fire double discharges (doublets) with very short intradoublet ISIs (up to a few ms). Each doublet is commonly followed by a prolonged post-doublet (interdoublet) ISI resulting from the AHP summation (Calvin and Schwindt, 1972). Two types of doublets have been observed: single and repetitive ones. Single doublets have been reported many times in cat and human motoneurons (e.g., Eccles and Hoff, 1932; Hoff and Grant, 1944; Denslow, 1948; Calvin and Schwindt, 1972; Kudina, 1974; Kudina and Churikova, 1990; Kirkwood and Munson, 1996; Piotrkiewicz et al., 2008; Stephenson and Maluf, 2010). Repetitive doublets have also been demonstrated in some of the reports above, but as a rule by the way; and only a few studies have directly addressed this topic (Bawa and Calancie, 1983; Kudina and Alexeeva, 1992; Kudina and Andreeva, 2010). However, a number of questions are still unanswered, in particular the question of whether there are certain muscles and (or) certain motoneurons for which repetitive doublet firing is likely to appear. This question will be taken up below based on an analysis of doublet firing in different limb and trunk muscles in healthy humans.

With regard to the origin of this surprising firing, currently, the short delayed depolarization (DD) appearing as a “hump” on the falling phase of an action potential, preceding the AHP (Granit et al., 1963; Kernell, 1964; Nelson and Burke, 1967; Kernell, 2006) and thus shortly increasing MN excitability is widely accepted as the mechanism responsible for *single* doublets in cat (Calvin and Schwindt, 1972; Kirkwood and Munson, 1996; see also Kernell, 2006), and human motoneurons (Kudina, 1974; Bawa and Calancie, 1983; Kudina and Alexeeva, 1992; Bawa and Lemon, 1993; Garland and Griffin, 1999; Piotrkiewicz et al., 2008; Stephenson and Maluf, 2010). As to the origin of *repetitive* doublets, the question still remains open. At first sight, one could assume that they have the same underlying mechanism like single doublets. However, as known from findings by Granit et al. (1963) and Calvin (1975), in cat motoneurons, the hump-DD changes systematically during rhythmic firing evoked by intracellular depolarization, becoming smaller with each successive spike. Therefore, as may be expected, in firing motoneurons the DD can reach the threshold only occasionally and generate single doublets only. Consequently, it might be suggested that the DD *alone* can hardly play a pivotal role in producing repetitive doublets.

In our recent studies (Kudina and Andreeva, 2010, 2013), we hypothesized that the mechanism underlying repetitive doublet firing in human motoneurons during voluntary muscle contraction is complex, including the hump-DD *as the primary determinant* that possibly becomes persistent due to a plateau potential probably activated in parallel with a common synaptic input. The assumption about a plateau potential role in repetitive doublet mechanisms was inferred from the comparison between essential characteristics of human motor unit (MU) repetitive doublet firing and properties of cat motoneuron firing in the presence of a plateau potential (e.g., Conway et al., 1988). If this assumption is true, then the question arises what are the DD characteristics which become persistent during repetitive doublet firing. The question will be considered below.

## Analysis of motoneuron doublet firing properties

In order to analyze the firing behavior of human motoneurons, MU recordings during voluntary muscle contractions were used. Five healthy volunteers (aged 46–62 years) took part in this investigation. All subjects gave written informed consent for the experimental procedures, which were approved by the local Ethics Committee and conformed to the Declaration of Helsinki. Six muscles were investigated: the trapezius, latissimus dorsi, triceps brachii, flexor carpi ulnaris, first dorsal interosseous, and tibialis anterior. The detailed information on the methods is given earlier (Kudina and Andreeva, 2010). Briefly, the potentials of single MUs were recorded using a bipolar needle electrode, amplified by an electromyograph DISA and stored on the magnetic tape for off-line analysis. During the experiments, subjects were asked to recruit a few MUs by slowly developing a gentle voluntary contraction of a muscle investigated, to maintain MU firing for a few minutes (using MU potential feedback), and then to relax. These ramp-and-hold contractions were repeated many times throughout the experiment. Before the recordings, the subjects were trained for approximately 15-20 min to search for MUs capable of firing double discharges. The MU potentials were transferred to a computer by an A/D converter with the sampling rate of 10 kHz. Action potentials of each MU were identified on the basis of their amplitude and waveform shape. The results of the computer identification were verified by visual inspection of single MU records by an experienced operator.

**Figure 1:**
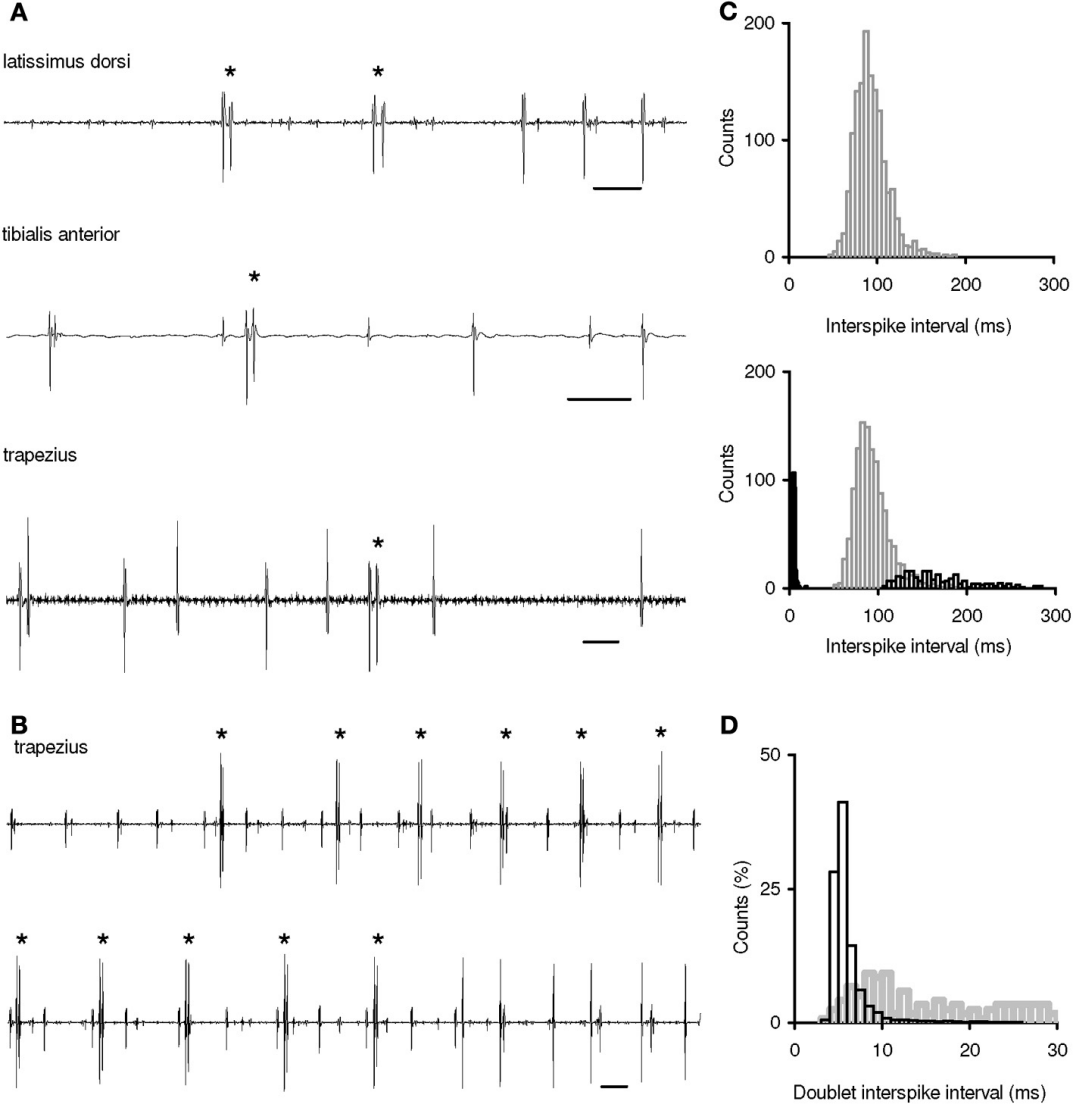
**Doublet firing in human motoneurons during voluntary muscle contractions. (A)** Examples of MU single doublets. Top, two initial doublets at MU recruitment; middle, a doublet among single-spike firing; bottom, a doublet at MU de-recruitment. **(B)** Examples of repetitive doublets at MU recruitment (two records of MU potentials are in succession). Doublets are marked by asterisks. Time bar: 50 ms. **(C)** Interspike interval (ISI) distributions of two trapezius MUs from the same experiment. Top, an MU displaying only single-spike firing (*n* = 1,418). Bottom, an MU displaying both single-spike firing and doublet firing; gray bars: single-spike ISIs (*n* = 1,193), black filled bars: intradoublet ISIs (*n* = 234), black open bars: interdoublet ISIs (*n* = 234). Bin width for intradoublet ISIs, 1 ms; for the rest, 5 ms. **(D)** MU intradoublet ISIs in various muscles; black bars: single and repetitive doublets in the trapezius, triceps brachii, and latissimus dorsi (*n* = 8,807); gray bars: single doublets in the flexor carpi ulnaris, first dorsal interosseous, and tibialis anterior (*n* = 107). Bin width, 1 ms.

A total of 228 MUs were identified; 96 MUs (42.1 %) displayed both single-spike and doublet firing, while the remaining ones were found to fire only more typical single discharges. Mean discharge rates of both MU groups were commonly in the range of 6–17 imp/s. Concerning the MU recruitment thresholds, note that all MUs were recorded at low-force contractions and, thus, belonged to the lowest-threshold part of motoneuron pools. Typical examples of single doublets are shown in Figure 1A. They were recorded at MU recruitment, among single-spike firing or at MU de-recruitment, i.e., the conditions of doublet occurrence in the muscle under study were consistent with previous observations in the literature. Repetitive doublets appeared typically in motoneurones at their recruitment, i.e., repetitive doublet trains were initiated in quiescent motoneurones rather than in firing ones (Figure 1B).

To obtain common characteristics of firing behavior of MUs with and without repetitive doublets, their ISI distributions were plotted (Figure 1C). MUs that did and did not express repetitive doublet firing showed similar ISI distributions during their single-spike firing. In contrast, during repetitive doublet firing, the MUs demonstrated ISI distributions in which the intradoublet ISIs formed a separate group of short ISIs. Interdoublet ISIs were typically in the range of the longest single-spike ISIs or even beyond them.

It was found that although single doublets were seen to occur in each of the six muscles investigated, repetitive doublets (discharge trains including of 3-110 doublets in succession, each of those followed by a prolonged interdoublet ISI) were observed in only three of them: in the trapezius, triceps brachii, and latissimus dorsi. As a result, these muscles exhibited the high doublet incidence estimated as the mean number of doublets per a doublet discharging MU. As to the flexor carpi ulnaris, first dorsal interosseous, and tibialis anterior none of these muscles demonstrated repetitive doublets and their doublet incidence was very low. This raises the question of whether there are any common traits for muscles firing or not firing repetitive doublets.

It appeared that repetitive doublets tended to be common in motoneurons from cervical spinal segments supplying the trapezius (C_2_–C_4_), the triceps brachii and latissimus dorsi (C_7_–C_8_), in contrast to those from more caudal spinal segments innervating the first dorsal interosseous and flexor carpi ulnaris (C_8_, Th_1_), and especially lumbar and sacral segments supplying the tibialis anterior (L_4_–L_5_, S_1_), in which only single doublets were scarcely recorded.

Further we compared intradoublet ISIs in two groups of muscles - generating or not generating repetitive doublets (Figure 1D). Although the ranges of pooled intradoublet ISIs in both groups were rather close (3.3–30.0 and 5.2–27.3 ms), their mean ISI was found to be significantly shorter in the muscles that displayed repetitive doublets (6.1 ± 2.4 vs. 15.2 ± 7.5 ms; *P* < 0.05). Moreover, motoneurons with repetitive doublets displayed the marked mode in the intradoublet ISI distribution in the range of 4.0–6.0 ms. In this range, about 70% of intradoublet ISIs have been occurred.

## Discussion

The present findings showed that among spinal motoneuronal pools a certain cranio-caudal gradient can be suggested in the capacity of motoneurons for repetitive doublets during voluntary muscle contractions. This suggestion is in line with previous data for the trapezius (Denslow, 1948; Kudina, 1974; Kudina and Alexeeva, 1992; Kudina and Andreeva, 2010; Stephenson and Maluf, 2010) as the muscle with frequent repetitive doublets, and the flexor carpi ulnaris (Kudina and Churikova, 1990), the first dorsal interosseous (Day et al., 1989) and the tibialis anterior (Andreassen and Rosenfalck, 1980), as the muscles without repetitive doublets. This point is in keeping with findings in other muscles: the flexor carpi radialis (C_6_–C_7_), the muscle in which long trains of repetitive doublets have been reported for the first time (Bawa and Calancie, 1983), in contrast to the rectus femoris (L_2_–L_4_) in which only occasional doublets have been observed (Kudina, 1974).

These differences in the prevalence of repetitive doublets between the motoneuron pools could be either due to differences in the properties of the DD and (or) to differences in motoneuron metabotropic inputs resulting in the activation of plateau potentials that is necessary, as was hypothesized, for keeping the sustained DD (Kudina and Andreeva, 2010).

Currently, in human experiments, there is no possibility of the reliable estimation of the strength of metabotropic inputs to a motoneuron pool. However, there is the possibility to estimate whether there are differences in properties of the DD; in particular *its effective duration* can be inferred from intradoublet ISI durations. According to the present data, mean intradoublet ISIs was found to be significantly shorter in the muscles that displayed repetitive doublets. Thus, the data allow concluding that the short duration of intradoublet ISIs can be used as a rather reliable *predictor* of whether or not a given motoneuron can fire repetitive doublets. Moreover, motoneurons firing repetitive doublets displayed the marked mode in the intradoublet ISI distribution (i.e., preferred intradoublet ISIs) in the range of 4.0–6.0 ms. It means that there were preferable moments of the appearance of extra-spikes creating doublets. Thus, it can be suggested that during repetitive doublets, the hump DD appears to be rather persistent. This stands in contrast to the hump disappearance during rhythmic firing found in acute animal experiments (see Introduction). Obviously, in humans, during a voluntary muscle contraction, the DD was sustained by a certain additional mechanism (possibly plateau potentials?).

## Conclusion

During discussions of mechanisms underlying motoneuron firing behavior, the AHP is commonly considered as one of key factors, whereas the DD is mentioned only in passing, if any. However, phenomenon of repetitive doublet firing gives evidence that under certain conditions of natural motor control (for instance, during some postural tasks) the DD, in common with the AHP, can contribute to control of the motoneuron firing behavior in healthy humans. In summary, we like to emphasize that although the properties of repetitive doublets, their underlying mechanisms and functional significance need further investigation, but even at present the obtained results indicate that the analysis of repetitive doublet firing can be a promising approach to investigations of certain cellular mechanisms of diseased motoneurone pool as the doublet phenomenon is significantly enhanced in a number of motoneuron diseases (e.g., Partanen, 1978; Rowiñska-Marciñska et al., 1999; Kleine et al., 2008; Piotrkiewicz et al., 2008; Weber et al., 2009).
